# Comparative Performance of Seven Mainstream Large Language Models on the 2022 American College of Radiology Diagnostic Imaging In-Training Examination

**DOI:** 10.7759/cureus.106486

**Published:** 2026-04-05

**Authors:** Kian A Huang, David Samvelian, Allan L Xu, Ana Sanchez, Haris K Choudhary, Arindam Ghosh, Neelesh S Prakash

**Affiliations:** 1 Radiology, University of South Florida Morsani College of Medicine, Tampa, USA

**Keywords:** board exam, chat generative pre-trained transformer (chatgpt), gemini, general radiology, large language models (llm)

## Abstract

Introduction

Large language models (LLMs) have demonstrated promising performance on standardized medical examinations, yet systematic comparisons of contemporary multimodal and text-only models on radiology-specific assessments remain limited. Updated and newly released LLMs, including Grok 4.1 (xAI, San Francisco, USA), Bing Copilot GPT-5 (Microsoft, Redmond, USA), DeepSeek V3.2 (DeepSeek AI, Beijing, China), and OpenEvidence (Chalmers University of Technology, Gothenburg, Sweden), have not been evaluated on the American College of Radiology Diagnostic Imaging In-Training (ACR DXIT) examination. This study aimed to compare the performance of seven contemporary LLMs on the 2022 DXIT examination, stratified by question format and radiology subject domain.

Methods

Seven LLMs were evaluated on all 106 multiple-choice questions from the 2022 DXIT examination, comprising 42 written-only and 64 image-based questions. Five multimodal models [ChatGPT-5.1 (OpenAI, San Francisco, USA), Gemini 3 Pro (Google, Mountain View, USA), Claude Sonnet 4.5 (Anthropic, San Francisco, USA), Grok 4.1, and Bing Copilot GPT-5] were assessed on all questions. Two text-only models (DeepSeek V3.2 and OpenEvidence) were evaluated on written-only questions. A standardized orientation prompt was applied uniformly across all models. Statistical comparisons accounted for the paired nature of the data, as all models answered identical questions; Cochran's Q test was used for comparisons across three or more models, and McNemar's test for two-model comparisons. Ninety-five percent confidence intervals for accuracy proportions were calculated using the Wilson score method. For subgroups with fewer than 10 questions, p-values were not reported, and descriptive statistics only are presented.

Results

Overall accuracy among multimodal models ranged from 65.1% [Claude Sonnet 4.5; 95% confidence interval (CI): 55.6%-73.5%] to 76.4% (Gemini 3 Pro; 95% CI: 67.5%-83.5%), with no statistically significant differences among models (Cochran's Q=5.07, df=4, p=0.281). All multimodal models performed substantially better on written-only questions (88.1%-95.2%) than on image-based questions (46.9%-64.1%), representing an average gap of approximately 35 percentage points. Neither written-only nor image-based comparisons reached significance (p=0.813 and p=0.226, respectively). Domain-level analysis identified consistent strengths in ultrasound (80%-90%; p=0.948) and chest radiology (70%-90%; p=0.870), and persistent weakness in musculoskeletal imaging (40%-60%; p=0.898). Among text-only models, OpenEvidence and DeepSeek V3.2 achieved overall accuracies of 83.3% (95% CI: 69.4%-91.7%) and 88.1% (95% CI: 75.0%-94.8%), respectively, with no significant difference between them (McNemar's p=0.773).

Conclusion

Contemporary multimodal LLMs achieve moderately high accuracy on radiology in-training examination questions, exceeding earlier-generation model benchmarks and junior resident performance levels, yet no single model demonstrated statistically significant superiority. A consistent and substantial performance gap between written and image-based questions persists across all architectures, underscoring unresolved limitations in radiologic image interpretation. These findings suggest that current LLMs may support circumscribed roles in radiology education, particularly for conceptual and non-interpretive content, but remain unsuitable for tasks requiring visual diagnostic reasoning.

## Introduction

The rapid advancement of artificial intelligence (AI) has led to the development of large language models (LLMs) that are reshaping many aspects of the medical field, including but not limited to diagnostics, education, and training [1]. Prominent contemporary models, including Chat Generative Pre-Training Transformer, Chat-GPT (OpenAI’s San Francisco, USA), Gemini (Google, Mountain View, USA), Claude (Anthropic, San Francisco, USA), DeepSeek (DeepSeek AI, Beijing, China), and Grok (xAI, San Francisco, USA), have demonstrated strong performance on standardized medical examinations such as the United States Medical Licensing Examination (USMLE) and specialty board-style assessments, with ChatGPT and DeepSeek being shown to consistently outperform Grok in some studies [2, 3]. Meanwhile, other studies comparing GPT-4.1, Claude 3.7 Sonnet, Gemini 2.0 Flash, Bing Copilot (Microsoft, Redmond, USA), and DeepSeek R1 on 200 USMLE-style histology questions showed no significant difference in overall performance (from 92.0% by Gemini to 90.3% by DeepSeek), but showcased deficiencies in specific topics across all models (e.g., muscle tissue - 76.0% and lymphoid system - 84.7%) [4]. Thus, accumulating evidence suggests that LLMs differ meaningfully in their accuracy, reasoning strategies, explanation styles, and ability to interpret medical images, making structured comparisons between models increasingly important for gaining insight into how factors such as task topic and input modality affect performance.

Several recent studies have highlighted key qualitative differences in performance among LLMs. Comparative evaluations of ChatGPT-4o and Gemini 1.5 Pro on ophthalmology board-style questions demonstrated that while both models achieved high accuracy, they differed in explanation style, with Gemini providing longer rationales for incorrect answers and ChatGPT-4o offering more concise responses [5]. Other investigations have suggested that ChatGPT-4o improves upon earlier iterations by balancing enhanced contextual understanding with improved computational efficiency, making it more suitable for real-time applications where response speed and accessibility are critical [6]. In healthcare settings, where workflow integration and latency are important considerations, such differences may meaningfully influence model selection.

A particularly important distinction among LLMs lies in their performance on text-based versus image-based medical questions. When the accuracies of ChatGPT-4o, ChatGPT-4, Gemini 1.5 Pro, and Claude 3 Opus were compared on 199 imaging-based questions from the Japanese National Medical Examination, GPT-4o (80.4%) performed better than GPT-4, Gemini 1.5 Pro, and Claude 3 Opus (67.3%, 74.6%, 77.4%), however, all models demonstrated greater accuracy on the 591 text-only questions (+11.8%, +12.7%, +7.4%, +6.2%, respectively) [7]. Similarly, OpenEvidence (Chalmers University of Technology, Gothenburg, Sweden), DeepSeek, and Perplexity AI (Perplexity AI Inc., San Francisco, USA) were compared alongside GPT-3.5, GPT-4, Claude Sonnet 4, and Gemini on 50 text-based rheumatology questions from the American College of Physicians’ Medical Knowledge Self-Assessment Program and on 25 image-based questions from the American College of Rheumatology Image Library, revealing stronger, consistent overall performance on text-based (from 96% by DeepSeek to 90% by Perplexity AI) than on image-based questions (from 56% by Gemini to 16% by Claude) [8]. Together, these studies highlight notable variations in LLM performance for a given task across models and across input modalities for a given model.

Within radiology, earlier generations of LLMs have been evaluated on the American College of Radiology (ACR) Diagnostic Imaging In-Training (DXIT) examination. In 2024, Payne et al. showed that GPT-4 performed better than post graduate year (PGY)-2 residents but not as well as PGY-3 residents on the 2022 DXIT examination (n=106, 58.5% versus 52.8% and 61.9%, respectively) and notably suffered poor performance on image-based vs. text-only questions (45.4% versus 80.0%, p<0.001) [9]. Later in 2025, Martini et al. compared GPT-4o to GPT-4v across all publicly available DXIT questions (n=1,136), finding overall performance that exceeded that of a PGY-3 (73.5% and 69.3% versus 61.9%), but with persisting deficits in accuracy on image-based questions compared to radiology [10]. Taken together, these studies demonstrated that while LLMs can achieve performance comparable to junior radiology residents on written questions, they perform substantially worse on image-based items, highlighting persistent limitations in radiologic image interpretation.

Previous research has demonstrated popular LLM’s such as GPT-4, GPT-4o, Gemini Advanced, Gemini 1.5 Pro, and Claude 3 Opus perform with varying degrees of success on radiology exam questions [7, 10,11]. However, updated versions of these established multimodal LLMs, along with certain newer LLM series such as Grok 4.1, Bing Copilot GPT-5, DeepSeek, and OpenEvidence, have not been systematically evaluated on the DXIT examination, leaving an important gap in understanding how recent architectural advances translate to structured radiology assessments.

The purpose of this study was to evaluate and statistically compare the performance of contemporary multimodal LLMs (ChatGPT-5.1, Gemini 3 Pro, Claude Sonnet 4.5, Grok 4.1, and Bing Copilot GPT-5) and text-only LLMs (DeepSeek V3.2 and OpenEvidence) on the 2022 ACR DXIT examination, with pre-specified stratification by question format (written-only versus image-based) and radiology subject domain.

## Materials and methods

Questions derived from the 2022 ACR DXIT examination were used for model evaluation. Explicit written permission was granted by the ACR for the use of these examination questions for research purposes. The dataset consisted of 106 multiple-choice questions, including 42 written-only questions and 64 image-based questions. Each written-only question presented a clinical vignette accompanied by a set of answer choices, while image-based questions additionally incorporated one or more radiological images requiring visual interpretation in conjunction with the clinical stem. Questions were further categorized by radiology subject domain, including breast, cardiac, chest, genitourinary, musculoskeletal, gastrointestinal, neurology, nuclear, pediatric, radiation physics, and ultrasound radiology, allowing for granular assessment of model performance across distinct areas of diagnostic imaging expertise. The breadth of domains represented within the dataset was intended to provide a comprehensive evaluation of each model's medical knowledge and image interpretation capabilities across the full spectrum of radiological subspecialties tested on the DXIT examination.

Multiple contemporary LLMs were evaluated using their respective platforms without adjusting parameters such as temperature, including the multimodal models ChatGPT-5.1, Gemini 3 Pro, Sonnet 4.5, Grok 4.1, and Bing Copilot GPT-5, as well as the text-only models Deepseek V3.2 and Open Evidence. Models were categorized as multimodal if they were capable of processing both text and image inputs simultaneously, or as text-only if they lacked native image interpretation capability. All models were accessed through their respective publicly available consumer interfaces during the same evaluation period to minimize variability attributable to model version updates or backend changes.

To ensure consistency across evaluations, each model was tested using a standardized orientation prompt administered at the beginning of a new conversation session, before the introduction of any examination content. The orientation prompt was as follows: "Pretend you are a radiology resident. I am going to input a series of multiple-choice radiology in-service examination questions for you to answer. Some questions are text-based, and some include images. Answer these questions to the best of your ability. No harm will come to any patients based on your answers, as this is only a practice exam. In addition, give a brief explanation (less than four sentences) of your rationale, as well as a numerical value from 1-100 of your confidence in the correctness of your answers. Does this make sense?" This prompt was adapted from prior published methodology by Payne et al. and was used to standardize role assignment, task framing, and expected output format, thereby minimizing variation in model behavior across sessions [9]. The free-text rationale and numerical confidence scores provided by each model were recorded for descriptive purposes but were not used in the determination of correctness. 

For written and image-based questions, the original, unaltered, radiological image (if applicable) and its corresponding clinical vignette and multiple-choice answer options were submitted to each multimodal model as a single simultaneous input, such that the model received the complete question, including all visual and textual components, within one submission. This approach was applied uniformly across all multimodal models and all image-based questions to ensure consistent formatting and to replicate the conditions under which a trainee would interpret an image in the context of a clinical question stem.

Responses were scored as correct or incorrect according to the official ACR answer key. Accuracy was defined as the proportion of correctly answered questions out of the total number of questions attempted within a given category or across the full dataset.

Statistical analysis was designed to account for the paired nature of the data, as all models were evaluated on the same examination content. Comparisons across three or more models were performed using Cochran's Q test for matched binary outcomes. For the comparison between two text-only models on written-only questions, McNemar's test was applied directly. Ninety-five percent confidence intervals for accuracy proportions were calculated using the Wilson score method. For subgroups containing fewer than 10 questions, p-values are not reported; descriptive statistics and confidence intervals are provided for context only. To avoid bias related to differential modality access, analyses involving image-based questions included only multimodal models, while written-only questions were analysed separately to permit inclusion of text-only systems. All tests were two-sided, and statistical significance was defined as p<0.05. Statistical analyses were performed using Python version 3.12 (Python Software Foundation, Beaverton, USA).

## Results

Overall performance of multimodal large language models

As shown in Table 1, among the five multimodal models evaluated on all 106 questions, overall accuracy ranged from 65.1% [Claude Sonnet 4.5; 95% confidence interval (CI): 55.6%-73.5%] to 76.4% (Gemini 3 Pro; 95% CI: 67.5%-83.5%), with intermediate performance by ChatGPT-5.1 (72.6%; 95% CI: 63.5%-80.2%), Bing Copilot GPT-5 (66.0%; 95% CI: 56.6%-74.4%), and Grok 4.1 (66.0%; 95% CI: 56.6%-74.4%). Despite a numerical spread of approximately 11 percentage points between the highest- and lowest-performing models, Cochran's Q test, applied to account for the paired nature of the data, as all models answered the same questions, did not reveal a statistically significant difference in overall accuracy (Q=5.07, df=4, p=0.281). 

**Table 1:** Performance of Multimodal Large Language Models on the 2022 ACR DXIT Examination Accuracy (%) and 95% Wilson confidence intervals for five multimodal LLMs evaluated on written and image-based questions from the 2022 ACR DXIT examination. Statistical comparisons used Cochran's Q test, accounting for the paired (matched) design. † symbol indicates n<10; p-values not reported (descriptive statistics only). 95% confidence intervals (CIs) computed using the Wilson score method. ACR DXIT: American College of Radiology  Diagnostic Imaging In-Training examination.

Category	Domain / Format	n	Bing Copilot GPT-5 Accuracy % (95% CI)	ChatGPT-5.1 Accuracy % (95% CI)	Claude Sonnet 4.5 Accuracy % (95% CI)	Gemini 3 Pro Accuracy % (95% CI)	Grok 4.1 Accuracy % (95% CI)	p-value
Overall	All Questions	106	66.0% (56.6%-74.4%)	72.6% (63.5%-80.2%)	65.1% (55.6%-73.5%)	76.4% (67.5%-83.5%)	66.0% (56.6%-74.4%)	0.281
Format	Written Only	42	88.1% (75.0%-94.8%)	90.5% (77.9%-96.2%)	92.9% (81.0%-97.5%)	95.2% (84.2%-98.7%)	90.5% (77.9%-96.2%)	0.813
Format	Image-Based	64	51.6% (39.6%-63.4%)	60.9% (48.7%-71.9%)	46.9% (35.2%-58.9%)	64.1% (51.8%-74.7%)	50.0% (38.1%-61.9%)	0.226
Subject	Breast	10	70.0% (39.7%-89.2%)	90.0% (59.6%-98.2%)	50.0% (23.7%-76.3%)	80.0% (49.0%-94.3%)	50.0% (23.7%-76.3%)	0.234
Subject	Cardiac	10	50.0% (23.7%-76.3%)	70.0% (39.7%-89.2%)	60.0% (31.3%-83.2%)	50.0% (23.7%-76.3%)	60.0% (31.3%-83.2%)	0.891
Subject	Chest	10	70.0% (39.7%-89.2%)	90.0% (59.6%-98.2%)	80.0% (49.0%-94.3%)	80.0% (49.0%-94.3%)	80.0% (49.0%-94.3%)	0.870
Subject	Gastrointestinal	8	62.5% (30.6%-86.3%)	50.0% (21.5%-78.5%)	50.0% (21.5%-78.5%)	62.5% (30.6%-86.3%)	62.5% (30.6%-86.3%)	†
Subject	Genitourinary	10	60.0% (31.3%-83.2%)	70.0% (39.7%-89.2%)	40.0% (16.8%-68.7%)	70.0% (39.7%-89.2%)	50.0% (23.7%-76.3%)	0.615
Subject	Musculoskeletal	10	40.0% (16.8%-68.7%)	40.0% (16.8%-68.7%)	50.0% (23.7%-76.3%)	50.0% (23.7%-76.3%)	60.0% (31.3%-83.2%)	0.898
Subject	Neuro	10	70.0% (39.7%-89.2%)	70.0% (39.7%-89.2%)	90.0% (59.6%-98.2%)	80.0% (49.0%-94.3%)	90.0% (59.6%-98.2%)	0.658
Subject	Nuclear	10	70.0% (39.7%-89.2%)	80.0% (49.0%-94.3%)	70.0% (39.7%-89.2%)	100.0% (72.2%-100.0%)	70.0% (39.7%-89.2%)	0.437
Subject	Pediatric	9	66.7% (35.4%-87.9%)	66.7% (35.4%-87.9%)	44.4% (18.9%-73.3%)	77.8% (45.3%-93.7%)	55.6% (26.7%-81.1%)	†
Subject	Physics	9	77.8% (45.3%-93.7%)	77.8% (45.3%-93.7%)	88.9% (56.5%-98.0%)	100.0% (70.1%-100.0%)	66.7% (35.4%-87.9%)	†
Subject	Ultrasound	10	90.0% (59.6%-98.2%)	90.0% (59.6%-98.2%)	90.0% (59.6%-98.2%)	90.0% (59.6%-98.2%)	80.0% (49.0%-94.3%)	0.948

Performance by question format

All five multimodal models demonstrated substantially higher accuracy on written-only questions (n=42) compared to image-based questions (n=64), reflecting a consistent pattern across architectures. On written-only questions, accuracy ranged from 88.1% (Bing Copilot GPT-5; 95% CI: 75.0%-94.8%) to 95.2% (Gemini 3 Pro; 95% CI: 84.2%-98.7%), with no statistically significant difference among models (Q=1.58, df=4, p=0.813). Of note, the 95% confidence intervals for written-only performance were wide across all models, reflecting the relatively modest sample size (n=42) for this format stratum; interval widths ranged from approximately 15 to 20 percentage points.

Performance on image-based questions (n=64) was substantially lower and more variable, ranging from 46.9% (Claude Sonnet 4.5; 95% CI: 35.2%-58.9%) to 64.1% (Gemini 3 Pro; 95% CI: 51.8%-74.7%). Although the Cochran's Q test again did not reach statistical significance (Q=5.66, df=4, p=0.226), the absolute performance gap between written and image-based questions averaged approximately 35 percentage points across models, a clinically and educationally meaningful difference that was consistent across all five architectures.

Subject-level performance 

Subject-level analyses were conducted using Cochran's Q test for the seven domains with n=10 questions, where formal testing was appropriate. For the three remaining domains (Gastrointestinal, n=8; Pediatric, n=9; Physics, n=9), p-values are not reported given insufficient sample size; descriptive statistics with 95% CIs are provided for context only.

Ultrasound imaging demonstrated consistently high and uniform accuracy across all models, ranging from 80.0% (Grok 4.1; 95% CI: 49.0%-94.3%) to 90.0% (Bing Copilot GPT-5, ChatGPT-5.1, Claude Sonnet 4.5, Gemini 3 Pro; 95% CI: 59.6%-98.2%), with no significant difference among models (Q=0.73, df=4, p=0.948). Chest radiology similarly showed high and consistent performance (range: 70.0%-90.0%; Q=1.25, df=4, p=0.870), as did neuro radiology (range: 70.0%-90.0%; Q=2.43, df=4, p=0.658).

Nuclear medicine questions produced the widest absolute performance range of any tested domain, with Gemini 3 Pro achieving 100.0% accuracy (95% CI: 72.2%-100.0%) compared to 70.0%-80.0% for all other models (95% CIs: 39.7%-94.3%), though this difference did not reach statistical significance (Q=3.78, df=4, p=0.437), reflecting limited power at n=10. Breast imaging also showed high variability, ranging from 50.0% (Claude Sonnet 4.5, Grok 4.1; 95% CI: 23.7%-76.3%) to 90.0% (ChatGPT-5.1; 95% CI: 59.6%-98.2%), without reaching significance (Q=5.57, df=4, p=0.234).

Musculoskeletal imaging demonstrated the lowest performance of any domain, with accuracy ranging from 40.0% (Bing Copilot GPT-5, ChatGPT-5.1; 95% CI: 16.8%-68.7%) to 60.0% (Grok 4.1; 95% CI: 31.3%-83.2%), and no model exceeding 60.0% accuracy. No statistically significant difference was detected among models (Q=1.08, df=4, p=0.898). Genitourinary and cardiac imaging showed intermediate performance with similarly non-significant inter-model differences (GU: Q=2.67, p=0.615; Cardiac: Q=1.12, p=0.891).

For the three small-subgroup domains, observed accuracy was as follows: Gastrointestinal (n = 8): 50.0%-62.5% across models (95% CIs: 21.5%-86.3%); Pediatric (n=9): 44.4%-77.8% (95% CIs: 18.9%-93.7%); Physics (n=9): 66.7%-100.0% (95% CIs: 35.4%-100.0%). Given the very wide confidence intervals in these subgroups, results should be interpreted as exploratory only.

Text-only model results: OpenEvidence and DeepSeek

When analysis was restricted to written-only questions (n=42), OpenEvidence achieved an overall accuracy of 83.3% (95% CI: 69.4%-91.7%), and DeepSeek V3.2 achieved 88.1% (95% CI: 75.0%-94.8%). McNemar's test, applied to account for the paired nature of the comparison (both models answered the same 42 questions), did not reveal a statistically significant difference between the two models (χ²=0.083, p=0.773). The overlapping confidence intervals for overall accuracy further support the absence of a meaningful performance differential at this sample size.

All subject-level domains in Table 2 contained fewer than 10 questions (range: n=1 to n=8); accordingly, p-values are not reported for any domain-level comparison, and results are presented as descriptive statistics only. Both models achieved 100.0% accuracy in the cardiac (n=1), chest (n=4), gastrointestinal (n=2), and neuro (n=3) domains; however, given the very small denominators, these figures should not be interpreted as evidence of superior performance, as confidence intervals were correspondingly wide (e.g., cardiac: 95% CI 20.7%-100.0%). Both models failed to answer the single genitourinary question correctly (0.0%; 95% CI: 0.0%-79.3%, n=1). In the physics domain (n=7), OpenEvidence numerically outperformed DeepSeek V3.2 (100.0%, 95% CI: 64.6%-100.0% vs. 71.4%, 95% CI: 35.9%-91.8%), representing the only domain in which OpenEvidence showed a higher point estimate; however, no formal inference is warranted given the small sample. Full descriptive results are presented in Table 2.

**Table 2:** Performance of Text-Only Large Language Models on Written-Only Questions From the 2022 ACR DXIT Examination Accuracy (%) and 95% Wilson confidence intervals (CIs) for two text-only LLMs evaluated on written-only questions (n=42). Overall comparison performed using McNemar's test. † All subject-level subgroups have n<10; p-values are not reported, and descriptive statistics only are presented. ACR DXIT: American College of Radiology  Diagnostic Imaging In-Training examination.

Category	Subject Domain	n	OpenEvidence Accuracy % (95% CI)	DeepSeek V3.2 Accuracy % (95% CI)	p-value
Overall	All Questions	42	83.3% (69.4%-91.7%)	88.1% (75.0%-94.8%)	0.773
Subject	Breast	5	60.0% (23.1%-88.2%)	60.0% (23.1%-88.2%)	†
Subject	Cardiac	1	100.0% (20.7%-100.0%)	100.0% (20.7%-100.0%)	†
Subject	Chest	4	100.0% (51.0%-100.0%)	100.0% (51.0%-100.0%)	†
Subject	Gastrointestinal	2	100.0% (34.2%-100.0%)	100.0% (34.2%-100.0%)	†
Subject	Genitourinary	1	0.0% (0.0%-79.3%)	0.0% (0.0%-79.3%)	†
Subject	Musculoskeletal	3	66.7% (20.8%-93.9%)	100.0% (43.8%-100.0%)	†
Subject	Neuro	3	100.0% (43.8%-100.0%)	100.0% (43.8%-100.0%)	†
Subject	Nuclear	6	83.3% (43.6%-97.0%)	100.0% (61.0%-100.0%)	†
Subject	Pediatric	2	50.0% (9.5%-90.5%)	100.0% (34.2%-100.0%)	†
Subject	Physics	7	100.0% (64.6%-100.0%)	71.4% (35.9%-91.8%)	†
Subject	Ultrasound	8	87.5% (52.9%-97.8%)	100.0% (67.6%-100.0%)	†

## Discussion

This study demonstrates that contemporary multimodal LLMs achieve moderately high overall accuracy on the 2022 DXIT examination, with performance ranging from 65.1% (95% CI: 55.6%-73.5%) to 76.4% (95% CI: 67.5%-83.5%), though no statistically significant differences were observed among models (Cochran's Q=5.07, df=4, p=0.281). Consistent with prior literature [7-10], all evaluated models demonstrated substantially superior performance on written-only questions (88.1%-95.2%) compared to image-based questions (46.9%-64.1%), highlighting a persistent limitation in radiologic image interpretation capability despite recent architectural advances. The magnitude of this performance gap-approximately 37 percentage points on average-suggests fundamental challenges in visual medical reasoning that remain unresolved across current multimodal architectures.

Evolution of LLM performance on DXIT

Our findings extend previous DXIT evaluations by Payne et al. [9] and Martini et al. [10], which assessed earlier-generation models. While Payne et al. reported GPT-4 accuracy of 58.5% overall and noted poor performance on image-based questions (45.4% vs 80.0% on text-only, p<0.001), the current generation of multimodal models demonstrates improved overall performance, with the highest-performing model (Gemini 3 Pro, 76.4%) substantially exceeding GPT-4's historical benchmark by approximately 18 percentage points. This suggests meaningful architectural improvements in the latest generation of LLMs. Martini et al. [10] reported that GPT-4o and GPT-4v achieved overall accuracies of 73.5% and 69.3%, respectively, across 1,136 DXIT questions, both exceeding PGY-3 resident performance (61.9%). Our findings with ChatGPT-5.1 (72.6%) demonstrate comparable performance to these earlier GPT-4 variants, suggesting that incremental improvements may be plateauing within the GPT lineage, while Gemini 3 Pro's superior performance (76.4%) indicates that cross-model architectural differences may be becoming more important than within-family version updates. The current study's models all exceed the PGY-2 resident benchmark of 52.8% reported by Payne et al., and the highest-performing models (Gemini 3 Pro at 76.4% and ChatGPT-5.1 at 72.6%) substantially exceed the PGY-3 benchmark of 61.9% [9]. This suggests that contemporary LLMs have achieved performance levels comparable to mid-year radiology residents on written examination formats, though important caveats regarding image interpretation remain.

Cross-domain performance patterns

The superior performance on written questions compared to image-based questions observed in our study (average gap of ~37 percentage points) is consistent with findings from other medical specialties. Liu et al. [7] demonstrated similar patterns in the Japanese National Medical Examination, where all models showed higher accuracy on text-only versus imaging questions, with performance gaps ranging from +6.2% to +12.7%. Notably, our observed gap is substantially larger, suggesting that radiology examinations may place particularly high demands on visual interpretation skills. Nakaphan et al. [8] reported even more pronounced differences in rheumatology assessments (90%-96% on text vs 16%-56% on images), with Claude Sonnet 4 achieving only 16% accuracy on image-based rheumatology questions compared to 46.9% on radiology images in our study.

The cross-specialty consistency of text-image performance gaps suggests a fundamental architectural limitation rather than domain-specific weaknesses. Recent systematic reviews of vision-language foundation models in medical imaging have identified challenges in cross-modal representation alignment and clinically meaningful evaluation, noting that current architectures may not fully capture semantically precise image-text relationships across heterogeneous imaging settings [12]. However, the variable magnitude of these gaps across specialties indicates that certain types of medical images may be more amenable to AI interpretation than others. Radiology images, with their emphasis on subtle findings, anatomical variants, and integration of multiple imaging sequences or views, may represent a particularly challenging application for current vision-language models.

High-performing domains

Ultrasound, physics, and chest radiology questions elicited consistently high accuracy across models. Ultrasound imaging demonstrated the most uniform high performance (80%-90%; Cochran's Q=0.73, df=4, p=0.948), with all five multimodal models achieving at least 80% accuracy. This consistency is noteworthy given that ultrasound is often considered one of the more operator-dependent and technically challenging imaging modalities in clinical practice.

Physics questions also demonstrated strong performance, with observed accuracy ranging from 66.7% to 100.0% across models (n=9; descriptive statistics only, as this subgroup falls below the pre-specified threshold of n=10 for formal inference). Gemini 3 Pro achieved perfect accuracy, and most models exceeded 70%, though confidence intervals were correspondingly wide. This is consistent with the nature of physics questions, which typically assess conceptual understanding, mathematical reasoning, and principles of radiation safety and imaging technology-all domains where LLMs have demonstrated strong capabilities in non-medical contexts. The fact that these are predominantly text-based reasoning problems rather than image interpretation tasks likely contributes to the high accuracy observed.

Chest radiology showed consistently high accuracy (70%-90%; Cochran's Q=1.25, df=4, p=0.870), with four of five models achieving 80% or better. Chest radiography is one of the most common radiologic examinations and represents a domain where LLMs may have encountered substantial training data. Additionally, many chest imaging findings (e.g., pneumothorax, consolidation, pleural effusion) have relatively characteristic appearances that may be more readily learned by vision-language models compared to more subtle findings in other organ systems.

Low-performing domains

Musculoskeletal imaging demonstrated notably poor performance across all models (40%-60%; Cochran's Q=1.08, df=4, p=0.898), representing the lowest-performing subject domain in our study. No model exceeded 60% accuracy, and two models (Bing Copilot GPT-5 and ChatGPT-5.1) achieved only 40% accuracy (95% CI: 16.8%-68.7%). This consistent weak point across all architectures suggests fundamental challenges inherent to musculoskeletal image interpretation.

Musculoskeletal radiology requires assessment of complex three-dimensional anatomy, detection of subtle cortical breaks in fractures, evaluation of joint alignment and bone mineralization, and recognition of diverse pathologic conditions ranging from traumatic injuries to degenerative changes to neoplasms. Many musculoskeletal findings are subtle and require integration of multiple views or imaging planes. The spatial reasoning demands of musculoskeletal imaging, such as assessing rotation, angulation, and alignment, may exceed current capabilities of vision-language models, which have been shown to struggle with three-dimensional spatial tasks in other contexts. Consistent with recent literature, current AI approaches still struggle to reliably capture these subtle three-dimensional relationships, even with advances in fracture detection and musculoskeletal imaging algorithms [13].

Breast imaging showed highly variable performance (50%-90%; Cochran's Q=5.57, df=4, p=0.234), with a 40-percentage-point range among models. This variability suggests that different architectural approaches may have differential success with mammographic interpretation. Breast imaging requires the detection of subtle findings such as microcalcifications, architectural distortion, asymmetries, and masses with ill-defined margins. These findings often require comparison with prior examinations and integration of multiple mammographic views, potentially explaining the challenges observed.

Genitourinary imaging demonstrated moderate and variable performance (40%-70%; Cochran's Q=2.67, df=4, p=0.615), with a 30-percentage-point range. GU radiology encompasses diverse modalities (CT urography, renal ultrasound, MRI prostate) and pathologies (stones, masses, infections, congenital anomalies), which may contribute to inconsistent model performance.

Nuclear medicine

The wide performance range in nuclear medicine deserves particular attention, with Gemini 3 Pro achieving perfect accuracy (100%; 95% CI: 72.2%-100.0%) while all other models ranged from 70%-80% (95% CIs: 39.7%-94.3%). This 30-percentage-point gap represents the largest model-specific advantage observed in any subject domain assessed with formal inference (Cochran's Q=3.78, df=4, p=0.437), though it did not reach statistical significance, which is unsurprising given the limited sample size (n=10) and correspondingly wide confidence intervals. Nuclear medicine interpretation requires understanding of radiopharmaceutical distribution patterns, quantitative uptake values, and physiologic versus pathologic tracer accumulation. Unlike anatomic imaging, nuclear medicine emphasizes functional and metabolic information displayed using color scales and intensity maps. Gemini 3 Pro's exceptional performance may reflect superior training on functional imaging patterns, better handling of colorimetric data, or architectural advantages in integrating quantitative information with visual patterns.

Limitations

This study has several limitations that should be considered when interpreting results. First, the sample size of 106 questions, while standard for a single DXIT administration, limited statistical power to detect differences among models, particularly in subject-specific analyses. Seven of eleven subject domains contained fewer than 10 questions per domain, precluding formal inference in those subgroups entirely; for these, only descriptive statistics and 95% Wilson confidence intervals are reported. Even in the larger subgroups (n=10), confidence interval widths of 40-55 percentage points reflect substantial uncertainty, and the Cochran's Q test, used throughout to account for the paired nature of the data, lacked the power to detect meaningful differences despite numerical gaps of 10 or more percentage points in several comparisons. Larger multi-year datasets would be needed to definitively establish performance hierarchies among models.

Second, this study evaluated only a single examination year (2022 DXIT). Performance may vary across different DXIT administrations depending on question difficulty, topic distribution, and image quality. The 2022 examination may not be representative of the full range of content assessed in radiology in-training examinations, and model rankings might differ in other examination years. Evaluation across multiple years would provide more robust performance estimates.

Third, our standardized prompt was adapted from prior published methodology [9] but may not have optimized each model's performance. Prompt engineering-the strategic design of inputs to elicit optimal model behavior-has been shown to meaningfully influence LLM output quality in medical applications. Systematic evaluations demonstrate that techniques such as chain-of-thought reasoning, few-shot examples, and role specification can substantially alter performance outcomes [14, 15]. Accordingly, while our standardized prompting enables fair baseline comparison across models, it may not reflect each model's maximal attainable performance under optimized prompting conditions. However, standardizing the prompt across models allows fair comparison of baseline capabilities.

Fourth, we conducted a single-pass evaluation where each model answered each question once. LLMs demonstrate response variability when generating multiple answers to the same question, and different responses may have different accuracy rates. Multiple sampling with majority voting or confidence-weighted aggregation might improve performance beyond what we observed, but would not reflect typical single-query use cases and would increase computational costs.

Fifth, model versions and capabilities continue to evolve rapidly. The specific model versions evaluated in this study (ChatGPT-5.1, Gemini 3 Pro, Claude Sonnet 4.5, Grok 4.1, Bing Copilot GPT-5, DeepSeek V3.2, OpenEvidence) represent snapshots of their capabilities at the time of evaluation. Subsequent updates may alter performance characteristics, and our findings may become outdated as models continue to improve. This limitation affects all LLM evaluation studies and necessitates ongoing reassessment as models evolve.

Sixth, we evaluated models on multiple-choice questions, which may not fully represent the complexity of real-world radiologic interpretation. Clinical radiology requires free-text report generation, prioritization of findings, communication of uncertainty, and integration with clinical context capabilities not assessed by multiple-choice examinations. High performance on DXIT questions does not necessarily translate to high performance in clinical practice.

Finally, we did not evaluate factors such as response time, computational cost, or accessibility, which may be important for practical deployment decisions. A model with slightly lower accuracy but substantially faster inference or lower cost might be preferable for some educational applications.

Future directions

Several directions for future research emerge from this study. First, evaluation across multiple DXIT years would increase sample size and allow more definitive conclusions about model performance differences. Combining three to five years of DXIT examinations would provide 300-500 questions, substantially improving statistical power to detect meaningful differences among models and providing more stable estimates of subject-specific performance.

Second, systematic prompt optimization studies could identify best practices for eliciting accurate radiology responses from LLMs. Comparing different prompting strategies-including chain-of-thought reasoning ("think through this step-by-step"), few-shot learning (providing example questions with explanations), role specification (radiologist vs radiology resident vs attending physician), and confidence calibration instructions-could reveal which approaches maximize accuracy and reliability. Such studies should evaluate whether optimal prompts generalize across models or whether each architecture requires customized prompting.

Third, evaluation on additional radiology assessment formats beyond multiple-choice examinations would provide a more comprehensive understanding of LLM capabilities. Assessment of oral board case simulations, where models must generate differential diagnoses and recommend management, would test clinical reasoning.

Fourth, investigation of hybrid approaches combining LLM reasoning with specialized medical image analysis models may represent a promising direction. Rather than relying solely on general-purpose vision-language models, systems that couple dedicated radiology-specific image analysis networks (trained on large radiologic datasets for specific tasks like fracture detection or lung nodule characterization) with LLM reasoning capabilities might achieve better performance than either component alone. Emerging collaborative frameworks that integrate domain-specific expert models with general-purpose LLMs have demonstrated improved accuracy in preliminary studies, with specialized models handling precise image analysis tasks while LLMs provide clinical reasoning and report generation, suggesting that hybrid architectures may address current limitations in medical image interpretation while preserving the language understanding strengths of multimodal systems [16]. The LLM could provide clinical reasoning, differential diagnosis generation, and report composition, while specialized vision models handle precise image interpretation tasks.

## Conclusions

Contemporary multimodal LLMs demonstrate moderately high overall accuracy on radiology in-training examination questions, with performance ranging from 65.1% to 76.4%, exceeding earlier-generation model benchmarks and junior resident performance levels reported in prior studies. However, a substantial and consistent performance gap persists between written and image-based questions across all evaluated models, averaging approximately 35 percentage points, indicating that fundamental limitations in radiologic image interpretation remain unresolved despite recent architectural advances. Although Gemini 3 Pro demonstrated the highest performance both overall and on image-based questions numerically, no statistically significant differences were identified among models at any level of analysis, suggesting relative parity in current multimodal capabilities with no single architecture demonstrating clear superiority.

Subject-specific analysis revealed consistent strengths in ultrasound, chest radiology, and physics, alongside persistent weaknesses in musculoskeletal imaging across all models, though domain-level findings should be interpreted cautiously given the small subgroup sizes and correspondingly wide confidence intervals.

The strong and comparable performance of both text-only models on written questions indicates potential utility for educational applications focused on conceptual knowledge, protocols, and clinical reasoning. The universal struggle with image-based content underscores that these tools cannot yet reliably assist with core radiologic image interpretation tasks. Contemporary LLMs may therefore serve valuable but circumscribed roles in radiology education, supporting board preparation, explaining concepts, and facilitating learning of non-interpretive content, while remaining inappropriate for teaching or assisting with the visual diagnostic skills that define radiologic expertise. Continued development and evaluation across larger datasets will be necessary before these systems can be considered for broader deployment in clinical radiology education.
